# Processed Food: Nutrition, Safety, and Public Health

**DOI:** 10.3390/ijerph192416410

**Published:** 2022-12-07

**Authors:** Tânia Gonçalves Albuquerque, Adriana Pavesi Arisseto Bragotto, Helena S. Costa

**Affiliations:** 1 Departamento de Alimentação e Nutrição, Instituto Nacional de Saúde Doutor Ricardo Jorge, I.P. Av. Padre Cruz, 1649-016 Lisboa, Portugal; 2 REQUIMTE-LAQV/Faculdade de Farmácia da Universidade do Porto, R. Jorge de Viterbo Ferreira 228, 4050-313 Porto, Portugal; 3 Faculdade de Engenharia de Alimentos, Universidade Estadual de Campinas, Cidade Universitária, Campinas 13083-000, SP, Brazil

## 1. Introduction

Food processing comprises the activities involved during the transformation of raw materials from different origins (vegetable, animal) until a final product is achieved that is suitable for human consumption [1]. Food processing was traditionally developed with a focus on the long-time storage and transport of foods, using techniques such as cooking, curing, and smoking. However, the effective reduction in spoilage and pathogenic microorganisms achieved with processing techniques such as pasteurization and other heat treatment technologies has also allowed processed foods to become safer. Later development of food processing has also included strategies to increase the palatability and production of indulgent products [2].

In recent decades, with the industrialization and globalization of food systems, food processing has evolved rapidly, contributing to an immense variety of foods subject to different types of processing, which above all, have different impacts on health. Excessive consumption of processed foods is often associated with the early development of non-communicable diseases, mainly because they have been recognized as containing high levels of salt, fat (saturated and “trans”), and sugar.

Consumers are increasingly looking for processed foods, mainly because they are practical, tasty, attractive, accessible, and affordable [3,4]. In the last thirty years, the processed food market has grown as never before; every day, “new” processed foods with different characteristics become available on the market [5]. Due to its high availability, accessibility and variety, its presence in the diet of the population of all age groups is inevitable.

Food processing has evolved profoundly and rapidly in the past decades. It is often related to potential negative consequences on the nutritional quality of food, and, in turn, on the population’s dietary patterns and the increase in noncommunicable diseases. However, food processing should not be seen as a problem for human nutrition. On the contrary, it played a key role in the evolution of humanity and civilizations, making food safer and more diverse. Furthermore, it is extremely important to extend the shelf life of foods or simply to make them edible [6].

## 2. Processed Foods and Nutrition

Processed foods are generally recognized as a source of salt, saturated fat, “trans” fatty acids, and sugar. An excessive intake of these nutrients is perceived as the leading reason for an increased risk in the development of some of the major worldwide public health concerns, such as obesity, diabetes type 2, cancer, and cardiovascular diseases.

Salt is an ingredient, condiment, and nutrient playing a central role in human nutrition. However, its excessive use is associated with public health concerns, namely hypertension. Over the years, there has been an increase in cases of hypertension, and it is estimated that this disease is the cause of 7.5 million deaths per year [7]. The World Health Organization (WHO) recommends a salt intake of less than 5 g/day for the prevention of cardiovascular diseases [7]. According to the Global Action Plan for the Prevention and Control of Noncommunicable Diseases (2013–2020), the WHO has set a goal of reducing salt intake by 30% [8].

Foods in their natural form usually contain sodium, but in most foods, it is present in low amounts. Sodium is normally added to foods in the form of salt. In developed countries, about 75–80% of salt is obtained by eating processed foods, 5–10% occurs naturally in foods, and the remaining 10–15% results from salt added during food preparation or at the table [7,9,10]. On the other hand, in developing countries, the salt used for seasoning or in sauces plays a much more important role.

From the point of view of the food industry, salt, in addition to the flavor it gives to foods, plays a crucial role in food preservation and processing [10]. On the other hand, for the consumer, salt is an important element for the flavor of food, in addition to suppressing other less appreciated flavors. When the amounts of salt are drastically reduced, the consumer usually rejects these foods.

Fat in foods has been one of the most studied nutrients in recent decades, with a concern not only in the amount ingested, but also in its quality and composition [11]. The type of fatty acids determines not only the physicochemical characteristics of the fat (such as resistance to rancidity), but also its nutritional properties and health effects. Among the different types of fatty acids, (saturated (SFA), monounsaturated (MUFA), polyunsaturated (PUFA), and “trans” (TFA)), those frequently associated with undesirable effects on the health of the population are SFA and TFA. An excessive intake of foods rich in this type of fatty acid was strongly related to an increased risk of developing cardiovascular disease, obesity, diabetes and cancer [12,13,14].

The European Strategy for the Prevention and Control of Noncommunicable Diseases (2012–2016) proposed to “eliminate “trans” fats in food and replace them with polyunsaturated fats” [15]. In addition, this specific objective is also a part of the European Action Plan for Food and Nutrition (2015–2020), whose general objective is to improve the management of the food system, the quality of food, and the nutritional status of the population, as well as to promote health and well-being [16].

Considering that the high consumption of free sugar by the world’s population in recent years has contributed to the achievement of daily energy values that exceed those recommended by health authorities and, consequently, to the increase in obesity rates, the WHO has issued guidance on sugar intake and strongly recommended that its consumption by children and adults be reduced to contribute less than 10% (preferably 5%) of the total calorie intake. Strategies such as educational campaigns, the taxation of beverages containing added sugars, restrictions in the advertising of foods and beverages with added sugar, and the reformulation of these products by the food industries, among others, have been adopted in some countries to achieve the goal of sugar reduction [17].

However, the power of sweet taste to induce consumption and to motivate behavior is profound, suggesting the importance of this sense for many species [18]. Although mainly used in foods due to its sweet taste, sugar has many other functions in food technology, including its role as a preservative, texture modifier, fermentation substrate, and bulking agent [19].

Concerning other nutrients, it is well-known that their contents can be destroyed or removed depending on the degree of processing. Some vitamins and minerals, for example, can be destroyed by heating or drying foods, while phytochemicals and fibers can be removed when peeling the outer layers of vegetables, fruits, and whole grains. On the other hand, certain processing techniques are employed to retain nutrients such as the quick freezing of fruits and vegetables after harvesting. Moreover, foods fortified with specific nutrients during processing have prevented deficiencies and their related health problems in certain populations [20].

## 3. Safety Aspects of Processed Foods

Foodborne diseases caused by pathogens, chemical substances, allergens, and physical contaminants remain a global public health challenge, since new threats are continuously emerging, while others are being controlled [21].

In order to lower the risk of foodborne pathogens or spoilage microorganisms, food processing techniques are employed to control microbial growth or inactivate microorganisms in food products [22]. The control of such microorganisms has evolved throughout human history to allow the production of safer foods via the application of physical or natural antimicrobials-based strategies [23].

The processing of food may also inactivate a range of chemical toxicants, including some natural toxins such as lectins and cyanogenic glycosides. Others, such as mycotoxins and metals, can be partially eliminated during the polishing of grains. On the other hand, some techniques commonly used to thermally process food (i.e., roasting, baking, frying, barbecuing) may generate carcinogenic substances such as acrylamide, furan, and polycyclic aromatic hydrocarbons. In addition, processed foods are also recognized for containing substances of deliberate use in food production, such as pesticides and additives [24].

Food processing may also impact the ability of proteins to cause the acquisition of allergic sensitization. Fermentation and hydrolysis, for example, may have the potential to reduce allergenicity to such an extent that symptoms will not be elicited [25].

## 4. Impacts on Public Health

The current pandemic of obesity and other noncommunicable diseases related to the population’s eating habits poses a serious threat to future well-being and economic prosperity worldwide. Currently, there is a new paradigm regarding the health status and quality of life of the population, since people live longer, but live with more comorbidities (diabetes, cardiovascular diseases, respiratory diseases, obesity, and oncological diseases). These diseases are often associated with early mortality and morbidity and have a significant impact on the national economy, mainly due to reduced productivity, increased absenteeism, and healthcare costs. It is estimated that women in the European Union spend almost a quarter (23%) of their lives in ill health; for men this figure is almost a fifth (19%) [26,27].

Noncommunicable diseases tend to be of long duration and are the result of a combination of genetic, physiological, environmental, and behavioral factors. The five ‘major’ noncommunicable diseases are cardiovascular diseases, diabetes, cancers, chronic respiratory diseases, and mental disorders.

According to the WHO, noncommunicable diseases kill 41 million people a year, representing 71% of all deaths worldwide. It is estimated that the total annual number of deaths from these diseases will increase to 55 million by 2030 if preventive measures are not taken, namely through the adoption of a healthy lifestyle, which includes a varied and balanced diet, as well as physical exercise and the prevention of excess weight.

## 5. Conclusions

Public health refers to preventing disease, prolonging life, and promoting physical, mental, and social wellbeing. Therefore, it is of the utmost importance to study the nutritional and safety aspects of processed foods and to deepen knowledge on the consumption of such foods to envisage the potential impact on public health.
